# Identifying Windows of Opportunity for Active Living and Healthy Eating Policies in Connecticut, 2016

**DOI:** 10.5888/pcd15.170331

**Published:** 2018-03-01

**Authors:** Anna E. Greer, Ann Knausenberger

**Affiliations:** 1 Department of Public Health, Sacred Heart University, Fairfield, Connecticut; 2 Department of Physical Therapy and Human Movement Science, Sacred Heart University, Fairfield, Connecticut

## Abstract

We examined the relative importance of 23 community issues among elected officials and health directors in Connecticut in 2016. For this cross-sectional study, 74 elected officials (40.7% response rate) and 47 health directors (62.7% response rate), who were purposively sampled, completed a questionnaire to rate their perceived importance of 23 community issues. Eight of these issues were related to active living, healthy eating, or obesity. We used χ^2^ tests to evaluate differences in responses. Compared with elected officials, health directors significantly more often perceived obesity, access to healthy groceries, poor nutrition, lack of pedestrian walkways, and pedestrian safety as important. Elected officials significantly more often than health directors perceived lack of good jobs, quality of public education, and cost of living as important. Health advocates should work with both groups to develop and frame policies to address both upstream (eg, jobs, education) and downstream (eg, healthy eating policies) determinants of obesity.

## Background

More than one-third (36.5%) of Americans are obese (1). Active living and healthy eating are associated with a reduced risk of obesity (2). Unhealthy diets and physical inactivity, however, are a problem in the United States (3,4).

State legislatures can create policies to support healthy eating and active living. For example, Kansas addressed healthy food access with policies to support farmers markets, including restrictions on liability and city fees (5). Massachusetts passed legislation that provides financial incentives to communities that pass high-density zoning regulations to support walkable, urban centers (6).

State policies require support from elected officials and other key stakeholders. Little research has examined the relative importance of healthy eating and active living issues at the state level. One study surveyed elected officials in Hawaii on the perceived importance of 23 community issues (7). Six of the issues were related to active living, but only an increase in vehicular traffic was recognized as relatively important. A follow-up study examined elected official’s perceptions of the same issues from 2007 to 2013 (8). The perceived importance of obesity increased over time, while perceived importance of increasing vehicular traffic, poorly planned development, and pedestrian safety declined.

Another study examined the perceived importance of active living, healthy eating, and obesity among legislators in Kansas (9). Obesity was rated second highest, after jobs. Healthy eating and active living issues were not deemed important. These findings suggest legislators might be unaware of how active living and healthy eating policies support obesity prevention.

Our study is the first peer-reviewed study to examine the relative importance of active living and healthy eating issues among elected state officials and municipal health directors. The findings highlight issues for which political support exists and ideas for how advocates of healthy eating and active living policies might better frame these issues (10). Identifying those issues is a crucial step in the policy development process (10). Previous research shows that advocating for healthy eating and active living policies can result in policy change in these areas (11).

### Study context

Connecticut is a relatively small state with a population of approximately 3.6 million residents. The median household income is $70,331, ranging from $29,313 in Hartford to $208,078 in Weston (12); the median household income in the United States is $53,889 (13). The percentage of residents reporting white race is 81% but ranges from 28% in Hartford to 96% in Litchfield (14); in the United States, 77% of the population self-reports white race (13).

One-fourth (26.0%) of Connecticut residents are obese. Racial/ethnic disparities exist in the prevalence of obesity: 37.7% of the black population, 30.3% of the Hispanic population, and 24.3% of the white population are obese (15). Overall, 21.8% of Connecticut residents meet national physical activity recommendations; in the United States, 20.1% meet these recommendations (3). In Connecticut, 32.0% of adults consume fruits and 26.0% consume vegetables fewer than 1 time daily; these percentages are 37.7% and 22.6%, respectively, in the United States (4).

Data on the distribution of political ideology among Connecticut officials are not available. To estimate this distribution, we weighted national-level data about Democratic and Republican political ideologies in 2016 (16) by the proportion of Democrats and Republicans in the Connecticut state legislature in 2016 (17). Using this formula, we estimated that 37.1% of elected officials in Connecticut were socially liberal, 27.6% socially moderate, and 33.8% socially conservative; 25.4% were fiscally liberal, 31.1% fiscally moderate, and 43.1% fiscally conservative in 2016.

## Gathering and Analyzing Data

We surveyed all elected officials at the state level (ie, senators and representatives) and all health directors at the municipal level in Connecticut. We used a census approach, whereby every person in the identified population was selected. In Connecticut, local health agencies are under the jurisdiction of the municipality they serve rather than the state commissioner (18). A list of all elected officials and health directors was available on the respective websites of the Connecticut General Assembly (19) and the Connecticut Department of Health (20). This led to a study population of 182 elected officials and 75 health directors. Survey participants were recruited via postal and electronic mail. Forty-seven health directors (62.7% response rate) and 74 elected officials (40.7% response rate) completed the study questionnaire from January through November 2016 for a total of 121 respondents.

Study materials sent via postal mail included a cover letter, an informed consent form, a study questionnaire, and a self-addressed postage-paid envelope. Study materials sent via electronic mail included a cover letter, an informed consent form, and a web link that directed respondents to SurveyMonkey to complete the questionnaire. All participants provided informed consent before participating in the study. All respondents were sent 2 follow-up reminders via electronic and postal mail. No incentives were offered. The study was framed as an opportunity for advocates to understand the community priorities of elected officials and health directors. The Sacred Heart University Institutional Review Board approved all study procedures.

For the questionnaire, we used a survey developed for a study examining policy makers’ perceptions of community issues in Hawaii (7). The questionnaire invited respondents to rate the importance of 23 issues in Connecticut (Table 1) on a 5-point Likert scale (1, not a problem at all; 3, may or may not be a problem; 5, problem of extreme importance). These items were recoded for analysis into important (score of 1 or 2) or not important (score of 3, 4, or 5). A response of 3 was grouped with a response of 4 and 5 because respondents who perceive that an issue “may or may not be a problem” would be unlikely to act to support policies for that issue. Because our study focused on identifying opportunities for policy change, we used potential for policy action as the guide for creating categories. Respondents were asked to indicate their political ideology for social issues and fiscal issues (conservative, moderate, liberal), and beliefs that government restrictions and tax increases should be used to protect health (never/rarely, sometimes, often/always) (Table 2).

**Table 1:** Community Issues Deemed Important, Overall and by Respondent Type, Connecticut, 2016^a^

Community Issue	All, No. (%) (n = 121)^b^	Respondent Type, No. (%)		χ^2^_1_ (*P* Value)
		Elected Official (n = 74)^b^	Health Director (n = 47)^b^	
**Obesity-related issues**				
Obesity	89 (74.2)	44 (59.5)	45 (97.8)	21.8 (<.001)
Increasing traffic	85 (71.4)	53 (72.6)	32 (69.6)	0.1 (.72)
Poorly planned development/sprawl	61 (50.4)	41 (55.4)	20 (42.6)	1.9 (.17)
Poor nutrition	56 (48.3)	28 (40.0)	28 (60.9)	4.8 (.03)
Access to healthy groceries	55 (45.5)	27 (36.5)	28 (59.6)	6.2 (.01)
Pedestrian safety	42 (35.9)	18 (25.7)	24 (51.1)	7.9 (.005)
Lack of pedestrian walkways	42 (35.6)	19 (26.8)	23 (48.9)	6.1 (.01)
Lack of recreational activities	24 (19.8)	13 (17.6)	11 (23.4)	0.6 (.41)
**Other health issues**				
Drug abuse	114 (94.2)	67 (90.5)	47 (100.0)	4.7 (.03)
Access to health care	62 (51.2)	32 (43.2)	30 (63.8)	4.9 (.03)
Climate change	59 (50.0)	32 (44.4)	27 (58.7)	2.3 (.13)
Tobacco	54 (46.2)	21 (13.6)	33 (21.4)	24.5 (<.001)
Pandemic influenza	41 (35.7)	8 (11.8)	33 (70.2)	41.4 (<.001)
**Social issues**				
Crime	72 (61.5)	37 (52.9)	35 (74.5)	5.5 (.02)
Quality of public education	66 (55.5)	49 (68.1)	17 (36.2)	11.7 (.001)
Homelessness	62 (53.0)	40 (57.1)	22 (46.8)	1.2 (.27)
**Economic issues**				
Lack of good jobs	100 (82.6)	69 (93.2)	31 (66.0)	14.9 (<.001)
High taxes	95 (78.5)	56 (75.7)	39 (83.0)	0.9 (.34)
Poverty	91 (75.8)	54 (74.0)	37 (78.7)	0.4 (.55)
Cost of living	86 (73.5)	57 (81.4)	29 (61.7)	5.6 (.02)
Lack of affordable housing	83 (69.2)	55 (75.3)	28 (59.6)	11.7 (.07)
**Government issues**				
Ethics in government	70 (57.9)	46 (62.2)	24 (51.1)	1.5 (.22)
Government response to natural disasters	24 (20.5)	12 (17.1)	12 (25.5)	1.2 (.27)

a We surveyed all elected officials at the state level (ie, senators and representatives) and all health directors at the municipal level in Connecticut. Response rates were 40.7% (74 of 182) among state elected officials and 62.7% (47 of 75) among municipal health directors.

b Not all respondents answered all questions. Percentages are based on the number of respondents who answered question.

**Table 2:** Self-Reported Political Ideology and Beliefs,^a^ Overall and by Type of Respondent, Connecticut, 2016^b^

Political Ideology	All, No. (%) (n = 121)^c^	Respondent Type, No. (%)		χ^2^_2_ (*P* Value)
		Elected Official (n = 74)^c^	Health Director (n = 47)^c^	
**Social issues**				
Liberal	48 (41.4)	34 (48.6)	14 (30.4)	3.9 (.14)
Moderate	39 (33.6)	20 (28.6)	19 (41.3)	
Conservative	29 (25.0)	16 (22.9)	13 (28.3)	
**Fiscal issues**				
Liberal	28 (24.1)	25 (35.7)	3 (6.5)	17.0 (<.001)
Moderate	28 (24.1)	10 (14.3)	18 (39.1)	
Conservative	60 (51.7)	35 (50.0)	25 (54.3)	
**Government restrictions should be used to protect health**				
Never/rarely	31 (27.0)	25 (36.2)	6 (13.0)	8.3 (.02)
Sometimes	66 (57.4)	33 (47.8)	33 (71.7)	
Often/always	18 (15.7)	11 (15.9)	7 (15.2)	
**Government tax increases should be used to protect health**				
Never/rarely	45 (38.5)	35 (50.0)	10 (21.3)	12.2 (.002)
Sometimes	55 (47.0)	24 (34.3)	31 (66.0)	
Often/always	17 (14.5)	11 (15.7)	6 (12.8)	

a Respondents were asked to indicate their political ideology for social issues and fiscal issues (conservative, moderate, liberal) and beliefs (never/rarely, sometimes, often/always) that government restrictions and tax increases should be used to protect health.

b We surveyed all elected officials at the state level (ie, senators and representatives) and all health directors at the municipal level in Connecticut. Response rates were 40.7% (74 of 182) among state elected officials and 62.7% (47 of 75) among municipal health directors.

c Not all categories add to column head because some respondents did not answer all questions. Percentages are based on the number of respondents who answered question. Column percentages in each category may not add to 100 because of rounding.

All data were loaded into SPSS version 24.0 (IBM Corp) for analysis. We used descriptive statistics to describe the data and χ^2^ tests to examine associations between 1) respondent type (elected official or health director) and perceived importance of community issues (yes or no), 2) respondent type and political ideology, 3) political ideology and perceived importance of community issues (yes or no). We ran χ^2^ goodness-of-fit tests to determine whether the distribution of elected officials who were socially and fiscally liberal, moderate, and conservative generated by our estimates was different from the distribution among elected officials who responded to our survey. We were unable to run a similar analysis for health directors because we could not find data on the political ideologies of health directors in Connecticut. Statistical significance was set at *P* <.05 for all tests.

## Findings

We found no significant difference between the distribution of social ideology of elected officials who participated in our study and our estimated distribution of elected officials in the Connecticut state legislature in 2016 (χ^2^_2_ = 4.7, *P* = .10). However, the proportion of fiscally liberal elected officials in our study (35.7%) was greater than the estimated proportion (20.9%) (χ^2^_2_ = 10.1, *P* = .007). A larger proportion of elected officials (35.7%) than health directors (6.5%) reported being fiscally liberal (χ^2^_2_ =17.0, *P* < .001) (Table 2). Health directors more often than elected officials supported using government restrictions (χ^2^_2_ = 8.3, *P* = .02) and tax increases (χ^2^_2_ =12.2, *P* = .002) to protect the public’s health.

Nine issues were deemed important by at least half of both elected officials and health directors (Table 1). The 3 issues of greatest importance to both groups were drug abuse, lack of jobs, and cost of living. Among the obesity-related issues, only obesity, poorly planned development/sprawl, and increasing traffic were identified as important by at least half of both groups.

A significantly greater proportion of health directors than elected officials deemed obesity, access to healthy groceries, poor nutrition, lack of pedestrian walkways, pedestrian safety, crime, pandemic influenza, drug abuse, tobacco, and access to health care as important. A significantly greater proportion of elected officials than health directors deemed quality of public education, lack of good jobs, and cost of living as important.

A significantly greater proportion of fiscally liberal than fiscally conservative respondents perceived climate change, quality of public education, poorly planned development/sprawl, access to health care, poverty, pedestrian safety, lack of affordable housing, access to healthy groceries, poor nutrition, and homelessness as important (Table 3). On the other hand, a significantly greater proportion of fiscally conservative respondents than fiscally liberal respondents perceived high taxes as important.

**Table 3:** Community Issues Deemed Important Among Elected Officials (n = 74) and Health Directors (n = 47), by Self-Reported Political Ideology^a^, Connecticut, 2016^b^

Community Issue	No. (%)^c^			χ^2^_2_ (*P* Value)
	Liberal	Moderate	Conservative	
**Social Ideology^d^**				
**Obesity-related issues**				
Obesity	35 (41.7)	27 (32.1)	22 (26.2)	0.2 (.91)
Increasing traffic	32 (40.0)	26 (32.5)	22 (27.5))	0.7 (.69)
Poorly planned development/sprawl	30 (52.6)	19 (33.3)	8 (14.0)	8.8 (.01)
Poor nutrition	30 (54.5)	17 (30.9)	8 (14.5)	8.6 (.01)
Access to healthy groceries	29 (56.9)	15 (29.4)	7 (13.7)	10.4 (.006)
Pedestrian safety	19 (46.3)	10 (24.4)	12 (29.3)	2.4 (.29)
Lack of pedestrian walkways	24 (58.5)	10 (24.4)	7 (17.1)	7.1 (.03)
Lack of recreational activities	11 (52.4)	6 (28.6)	4 (19.0)	1.3 (.52)
**Other health issues**				
Drug abuse	45 (41.3)	36 (33.0)	28 (25.7)	0.5 (.76)
Access to health care	31 (54.4)	16 (28.1)	10 (17.5)	8.1 (.02)
Climate change	30 (53.6)	19 (33.9)	7 (12.5)	11.3 (.004)
Tobacco	26 (49.1)	18 (34.0)	9 (17.0)	3.9 (.14)
Pandemic influenza	13 (32.5)	14 (35.0)	13 (32.5)	2.4 (.30)
**Social issues**				
Crime	27 (38.0)	25 (35.2)	19 (26.8)	0.9 (.65)
Quality of public education	32 (52.5)	17 (27.9)	12 (19.7)	6.8 (.03)
Homelessness	31 (50.8)	18 (29.5)	12 (19.7)	4.9 (.09)
**Economic issues**				
Lack of good jobs	44 (46.3)	30 (31.6)	21 (22.1)	5.5 (.06)
High taxes	33 (35.9)	32 (34.8)	27 (29.3)	6.8 (.03)
Poverty	42 (48.8)	25 (29.1)	10 (22.1)	7.1 (.03)
Cost of living	34 (40.0)	31 (36.5)	20 (23.5)	1.2 (.55)
Lack of affordable housing	38 (48.1)	22 (27.8)	19 (24.1)	5.2 (.07)
**Government issues**				
Ethics in government	26 (38.8)	26 (38.8)	15 (22.4)	2.0 (.38)
Government response to natural disasters	11 (47.8)	9 (39.1)	3 (13.0)	2.2 (.34)
**Fiscal Ideology^e^**				
**Obesity-related issues**				
Obesity	20 (23.8)	22 (26.2)	42 (50.0)	0.6 (.75)
Increasing traffic	22 (27.5)	19 (23.8)	39 (48.8)	1.3 (.53)
Poorly planned development/sprawl	21 (36.8)	12 (21.1)	24 (42.1)	9.9 (.007)
Poor nutrition	19 (34.5)	19 (34.5)	17 (30.9)	17.6 (<.001)
Access to healthy groceries	19 (33.3)	19 (37.2)	15 (29.4)	19.0 (<.001)
Pedestrian safety	13 (31.7)	11 (26.8)	17 (41.5)	3.0 (.22)
Lack of pedestrian walkways	13 (31.7)	14 (34.1)	14 (34.1)	7.2 (.03)
Lack of recreational activities	4 (19.0)	7 (33.3)	10 (47.6)	1.3 (.53)
**Other health issues**				
Drug abuse	26 (23.9)	27 (24.8)	56 (51.4)	0.4 (.82)
Access to health care	21 (36.8)	16 (28.1)	20 (35.1)	14.2 (.001)
Climate change	21 (37.5)	19 (33.9)	16 (28.6)	25.8 (<.001)
Tobacco	12 (22.6)	17 (32.1)	24 (45.3)	3.4 (.18)
Pandemic influenza	7 (17.5)	12 (30.0)	21 (52.5)	2.0 (.36)
**Social issues**				
Crime	17 (23.9)	19 (26.8)	35 (49.3)	0.7 (.69)
Quality of public education	22 (36.1)	16 (26.2)	23 (37.7)	13.6 (.001)
Homelessness	22 (36.1)	19 (31.1)	20 (32.8)	19.1 (<.001)
**Economic issues**				
Lack of good jobs	25 (26.3)	23 (24.2)	47 (49.5)	1.5 (.46)
High taxes	16 (17.4)	23 (25.0)	53 (57.6)	11.5 (.003)
Poverty	28 (32.6)	23 (26.7)	35 (40.7)	17.7 (<.001)
Cost of living	19 (22.4)	25 (29.4)	41 (48.2)	4.8 (.09)
Lack of affordable housing	26 (32.9)	21 (26.6)	32 (40.5)	14.5 (.001)
**Government issues**				
Ethics in government	18 (26.9)	13 (19.4)	36 (53.7)	2.0 (.35)
Government response to natural disasters	7 (30.4)	5 (26.1)	10 (43.5)	0.9 (.64)

a Respondents were asked to indicate their political ideology for social issues and fiscal issues (conservative, moderate, liberal).

b We surveyed all elected officials at the state level (ie, senators and representatives) and all health directors at the municipal level in Connecticut. Response rates were 40.7% (74 of 182) among state elected officials and 62.7% (47 of 75) among municipal health directors.

c Not all respondents answered all questions. Percentages are based on the number of respondents who answered question. Row percentages may not add to 100 because of rounding.

d For social ideology among elected officials and health directors who answered question (n = 116), 29 self-reported as liberal, 39 as moderate, and 48 as conservative.

e For fiscal ideology among elected officials and health directors who answered question (n = 116), 60 self-reported as liberal, 28 as moderate, and 28 as conservative.

A significantly greater proportion of socially liberal respondents than socially conservative respondents perceived climate change, quality of public education, poorly planned development/sprawl, access to health care, poverty, pedestrian safety, access to healthy groceries, and poor nutrition as important. Compared with socially liberal respondents, socially conservative respondents more often perceived high taxes as important.

## Opportunities for Application of Findings

Elected officials and fiscally conservative respondents were less supportive of tax increases to protect health and more concerned about high taxes than health directors and fiscally liberal respondents. The elected officials in our study were more fiscally liberal than the body of all elected officials in Connecticut, indicating that support for tax increases to protect health is likely even lower in Connecticut than estimated by our study. Our findings suggest that health advocates will likely find resistance when advocating for policies that require a large amount of funding for policy implementation. An alternative to an increase in taxes is a collaboration of community partners that contribute funds. For example, the Pennsylvania Fresh Food Financing Initiative was a public–private partnership that supported access to healthy food in that state (21). Pennsylvania provided a $30 million grant and the Reinvestment Fund contributed $145 million; these funds were used to provide loans to attract grocery stores to communities in need of access to healthy food (22).

Our findings also indicate that socially and fiscally liberal respondents perceived active living and healthy eating issues as more important than did socially and fiscally conservative respondents. Advocates must reframe their arguments for active living and healthy eating policies to identify how these policies can support personal and public cost savings. For example, a tax on sugar-sweetened beverages could reduce soda consumption while raising revenue for states (23).

Obesity was identified as important by at least half of both elected officials and health directors. Policies, however, cannot be developed to ban or reduce obesity. Rather, policies that support active living and healthy eating can enable people to achieve a healthy weight. For example, North Carolina designated funds to develop a light rail system in Charlotte; use of this system was associated with a reduction in the odds of residents becoming obese (24). Previous studies identified limited awareness among elected officials about the connection between obesity and active living and healthy eating policies (7,9). Health advocates might share information on resources such as the State Legislative and Regulatory Action to Prevent Obesity and Improve Nutrition and Physical Activity (25) with elected officials and health directors, so that people who are developing policy have clear examples from which to work.

Elected officials in our study were more concerned than health directors with public education, lack of good jobs, and cost of living. Social determinants, or conditions in which people are born and live, likely contribute most to the growing prevalence of obesity (2). Opportunities exist for elected officials and health directors to work together to develop policies that address issues of concern for both groups: upstream social determinants and downstream active living and healthy eating initiatives. For example, in Bridgeport, Connecticut, a zoning regulation was changed to allow food entrepreneurs to prepare healthy food products in church kitchens before selling them locally at farmers markets (26). Before this change, food production could occur only in more expensive commercial kitchens, limiting opportunities for healthy food production and sale by lower-income residents. Policies like this at the state level have the potential to affect both job opportunities and access to healthy food.

The only active living issues deemed as important by at least half of both elected officials and health directors were increasing traffic and poorly planned development/sprawl. A study in 2007 identified increasing traffic as important among Hawaii elected officials (7), but by 2013 the importance of increasing traffic had declined (8). Policies to reduce traffic and poorly planned development/sprawl should be pursued immediately in Connecticut because support exists from both elected officials and health directors at this time. The presence of sidewalks, an issue of importance to health directors, or increased funding for public transportation could support active transportation (27,28) and reduce traffic (29).

The 3 issues of greatest importance to both groups were drug abuse, lack of good jobs, and cost of living. These are vital issues in Connecticut (30,31). The proportion of persons with past-year illicit drug abuse was higher in Connecticut (2.9%) than in the United States (2.6%) (30). The cost of living in Connecticut is 29% higher than it is for the average US household (31). The proportion of persons in the Connecticut labor force (67.2%) is higher than the national average (63.3%) (32). However, employment is an important determinant of well-being (2), supporting Connecticut leaders’ concern for job opportunities.

Our findings indicate that windows of opportunity currently do not exist for healthy eating policies unless they are linked to obesity or other areas of alignment. A study in 2013 (9) found that elected officials in Kansas did not view healthy eating issues as relatively important. Because health directors in our study more often than elected officials perceived poor nutrition and access to healthy groceries as important, health directors should work with elected officials to communicate information on the links between healthy eating policies and obesity prevention.

## Additional Considerations

Our study has several limitations. The study is cross-sectional and therefore does not allow causal inferences. Questionnaire responses were self-reported, so they could have been affected by social desirability bias. Our findings have limited generalizability and could differ in a subsequent election cycle. The issues addressed in the questionnaire ranged in specificity. For example, education could influence myriad outcomes. Too few sidewalks, on the other hand, is a specific barrier to promoting active living. Our study is strengthened by the inclusion of a questionnaire used in 3 previous studies (7–9), which allowed for comparisons across studies and states. In addition, our analyses of how the political ideologies of elected officials in our study align with all Connecticut elected officials allow readers to better determine generalizability.

Our findings indicate that active living and healthy eating issues are priority areas for health directors but that elected officials are more concerned with social determinants of health. Health advocates should work with health directors and elected officials to develop policies that address both upstream social determinants (eg, jobs, education) and downstream behavioral supports (eg, physical activity and healthy eating policies) that act as determinants of obesity. Health advocates will have more success if proposed policies do not increase taxes.
